# 2-(3,5-Di-*tert*-butyl-4-hydroxy­benzyl­sulfan­yl)nicotinic acid

**DOI:** 10.1107/S1600536808026056

**Published:** 2008-08-16

**Authors:** Shahirah Mansor, Wagee A. Yehye, Azhar Ariffin, Noorsaadah Abdul Rahman, Seik Weng Ng

**Affiliations:** a Department of Chemistry, University of Malaya, 50603 Kuala Lumpur, Malaysia

## Abstract

Two mol­ecules of the title compound, C_21_H_27_NO_3_S, are disposed about a center of inversion, generating an O—H⋯O hydrogen-bonded dimer.

## Related literature

For the applications of hindered phenol-based anti­oxidants, see: Kim & Lee (2003 ▶); Um & Lee (2005 ▶).
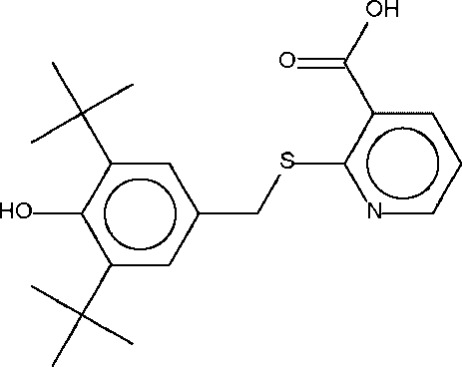


## Experimental

### 

#### Crystal data

C_21_H_27_NO_3_S
*M* *_r_* = 373.50
Triclinic, 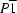

*a* = 5.6305 (1) Å
*b* = 9.3489 (2) Å
*c* = 18.8749 (3) Å
α = 85.505 (1)°
β = 89.453 (1)°
γ = 87.834 (1)°
*V* = 989.77 (3) Å^3^
*Z* = 2
Mo *K*α radiation
μ = 0.18 mm^−1^
*T* = 100 (2) K
0.25 × 0.15 × 0.05 mm

#### Data collection

Bruker SMART APEX diffractometer
Absorption correction: multi-scan (*SADABS*; Sheldrick, 1996 ▶) *T*_min_ = 0.956, *T*_max_ = 0.991
12688 measured reflections
4507 independent reflections
3746 reflections with *I* > 2σ(*I*)
*R*_int_ = 0.028

#### Refinement

*R*[*F* ^2^ > 2σ(*F* ^2^)] = 0.042
*wR*(*F* ^2^) = 0.127
*S* = 1.18
4507 reflections
239 parameters
1 restraint
H atoms treated by a mixture of independent and constrained refinement
Δρ_max_ = 0.75 e Å^−3^
Δρ_min_ = −0.71 e Å^−3^

### 

Data collection: *APEX2* (Bruker, 2007 ▶); cell refinement: *SAINT* (Bruker, 2007 ▶); data reduction: *SAINT*; program(s) used to solve structure: *SHELXS97* (Sheldrick, 2008 ▶); program(s) used to refine structure: *SHELXL97* (Sheldrick, 2008 ▶); molecular graphics: *X-SEED* (Barbour, 2001 ▶); software used to prepare material for publication: *publCIF* (Westrip, 2008 ▶).

## Supplementary Material

Crystal structure: contains datablocks global, I. DOI: 10.1107/S1600536808026056/tk2295sup1.cif

Structure factors: contains datablocks I. DOI: 10.1107/S1600536808026056/tk2295Isup2.hkl

Additional supplementary materials:  crystallographic information; 3D view; checkCIF report

## Figures and Tables

**Table 1:** Hydrogen-bond geometry (Å, °)

*D*—H⋯*A*	*D*—H	H⋯*A*	*D*⋯*A*	*D*—H⋯*A*
O1—H1*o*⋯O2^i^	0.85 (1)	1.79 (1)	2.640 (2)	179 (3)

Symmetry code: (i) 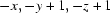.

**Fig. 1.**
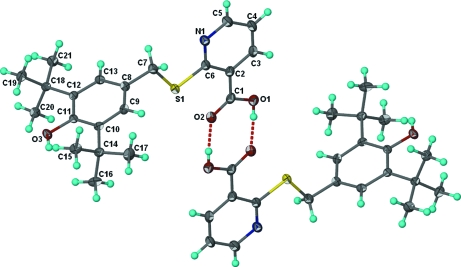
Thermal ellipsoid plot (Barbour, 2001) of the hydrogen-bonded dimeric structure of (I) drawn at the 70% probability level. Dashed lines denote the hydrogen bonds. Hydrogen atoms are drawn as spheres of arbitrary radius.

## supplementary crystallographic information

### Comment

The compound (I, Fig. 1) is the precursor for the synthesis of hindered phenol-based antioxidants. Phenol-based antioxidants and their derivative have applications in industries such as pharmaceutical, textiles, plastics, polymers, oils, pesticides,dyestuffs, explosives, fluorescent-brightening industries (Kim & Lee, 2003; Um & Lee, 2005). Molecules are connected into centrosymmetric dimers via the eight- membered {OCOH}_2_ synthon (Table 1). The hydroxyl-H projects between the two sterically hindered aromatic rings and is therefore precluded from forming a hydrogen bonding interaction.

### Experimental

2-Mercaptonicotinic acid (1.50 g, 1 mmol), 2,6-di-*t*-butylphenol (2.00 g, 1 mmol) and paraformaldehyde (0.291 g,1 mmol) were intimately ground into a powder and to this was added di-*n*-butylamine (0.09 ml). The slurry was heated to 373–383 K and after an hour, this solidified. The solid was purified by column chromatography, with chloroform as solvent, to give two products, one of which was the expected acid, (I), and the other, the di-*n*-butylammonium salt of the acid.

### Refinement

Carbon-bound H-atoms were placed in calculated positions (C—H 0.95 to 0.99 Å) and were included in the refinement in the riding model approximation, with *U*(H) set to 1.2–1.5*U*(C). The acid H-atom was located in a difference Fourier map, and was refined with a distance restraint of O–H 0.84±0.01 Å; its temperature factor were freely refined. The hydroxy H-atom was placed in a chemically sensible position, with a distance of more than 2 Å from the neighboring methyl H-atoms. The C–O–H fragment is then perpendicular to the aromatic ring.

### Crystal data

**Table:**

C_21_H_27_NO_3_S	*Z* = 2
*M**_r_* = 373.50	*F*_000_ = 400
Triclinic, *P*1	*D*_x_ = 1.253 Mg m^−^^3^
Hall symbol: -P 1	Mo *K*α radiation λ = 0.71073 Å
*a* = 5.6305 (1) Å	Cell parameters from 3920 reflections
*b* = 9.3489 (2) Å	θ = 2.2–28.4º
*c* = 18.8749 (3) Å	µ = 0.18 mm^−^^1^
α = 85.505 (1)º	*T* = 100 (2) K
β = 89.453 (1)º	Prism, colorless
γ = 87.834 (1)º	0.25 × 0.15 × 0.05 mm
*V* = 989.77 (3) Å^3^	

### Data collection

**Table:**

Bruker SMART APEX diffractometer	4507 independent reflections
Radiation source: fine-focus sealed tube	3746 reflections with *I* > 2σ(*I*)
Monochromator: graphite	*R*_int_ = 0.028
*T* = 100(2) K	θ_max_ = 27.5º
ω scans	θ_min_ = 1.1º
Absorption correction: Multi-scan(SADABS; Sheldrick, 1996)	*h* = −7→7
*T*_min_ = 0.956, *T*_max_ = 0.991	*k* = −9→12
12688 measured reflections	*l* = −24→24

### Refinement

**Table:**

Refinement on *F*^2^	Secondary atom site location: difference Fourier map
Least-squares matrix: full	Hydrogen site location: inferred from neighbouring sites
*R*[*F*^2^ > 2σ(*F*^2^)] = 0.042	H atoms treated by a mixture of independent and constrained refinement
*wR*(*F*^2^) = 0.127	*w* = 1/[σ^2^(*F*_o_^2^) + (0.0642*P*)^2^ + 0.2102*P*] where *P* = (*F*_o_^2^ + 2*F*_c_^2^)/3
*S* = 1.18	(Δ/σ)_max_ = 0.001
4507 reflections	Δρ_max_ = 0.75 e Å^−^^3^
239 parameters	Δρ_min_ = −0.71 e Å^−^^3^
1 restraint	Extinction correction: none
Primary atom site location: structure-invariant direct methods	

### Special details

**Table:**

Geometry. All e.s.d.'s (except the e.s.d. in the dihedral angle between two l.s. planes) are estimated using the full covariance matrix. The cell e.s.d.'s are taken into account individually in the estimation of e.s.d.'s in distances, angles and torsion angles; correlations between e.s.d.'s in cell parameters are only used when they are defined by crystal symmetry. An approximate (isotropic) treatment of cell e.s.d.'s is used for estimating e.s.d.'s involving l.s. planes.

### Fractional atomic coordinates and isotropic or equivalent isotropic displacement parameters (Å^2^)

**Table:**

	*x*	*y*	*z*	*U*_iso_*/*U*_eq_	
S1	0.58009 (7)	0.68988 (5)	0.60316 (2)	0.01722 (13)	
O1	0.0857 (2)	0.63037 (14)	0.42620 (6)	0.0211 (3)	
H1O	−0.014 (4)	0.566 (2)	0.4379 (14)	0.053 (8)*	
O2	0.2215 (2)	0.56848 (14)	0.53598 (6)	0.0222 (3)	
O3	0.8816 (2)	0.54828 (13)	0.92906 (6)	0.0161 (3)	
H3O	0.7848	0.4934	0.9503	0.024*	
N1	0.7549 (3)	0.87163 (16)	0.50351 (7)	0.0177 (3)	
C1	0.2358 (3)	0.64038 (19)	0.47879 (9)	0.0184 (4)	
C2	0.4212 (3)	0.74553 (19)	0.46400 (8)	0.0176 (4)	
C3	0.4393 (3)	0.8192 (2)	0.39715 (9)	0.0203 (4)	
H3	0.3316	0.8015	0.3607	0.024*	
C4	0.6135 (3)	0.9179 (2)	0.38393 (9)	0.0214 (4)	
H4	0.6284	0.9689	0.3386	0.026*	
C5	0.7661 (3)	0.9404 (2)	0.43878 (9)	0.0203 (4)	
H5	0.8856	1.0087	0.4299	0.024*	
C6	0.5857 (3)	0.77544 (19)	0.51655 (8)	0.0165 (3)	
C7	0.8320 (3)	0.7718 (2)	0.64204 (8)	0.0171 (3)	
H7A	0.9800	0.7489	0.6159	0.021*	
H7B	0.8070	0.8775	0.6397	0.021*	
C8	0.8498 (3)	0.71171 (19)	0.71821 (8)	0.0149 (3)	
C9	0.9743 (3)	0.58333 (19)	0.73576 (8)	0.0152 (3)	
H9	1.0514	0.5350	0.6990	0.018*	
C10	0.9903 (3)	0.52279 (18)	0.80543 (8)	0.0131 (3)	
C11	0.8749 (3)	0.59945 (18)	0.85839 (8)	0.0126 (3)	
C12	0.7483 (3)	0.73030 (18)	0.84288 (8)	0.0126 (3)	
C13	0.7379 (3)	0.78302 (18)	0.77173 (8)	0.0148 (3)	
H13	0.6514	0.8706	0.7595	0.018*	
C14	1.1234 (3)	0.37706 (18)	0.82171 (8)	0.0151 (3)	
C15	1.3288 (3)	0.38746 (19)	0.87432 (9)	0.0184 (4)	
H15A	1.4089	0.2930	0.8832	0.028*	
H15B	1.2653	0.4196	0.9192	0.028*	
H15C	1.4426	0.4564	0.8541	0.028*	
C16	0.9495 (3)	0.2627 (2)	0.85004 (10)	0.0213 (4)	
H16A	0.8223	0.2563	0.8155	0.032*	
H16B	0.8807	0.2896	0.8952	0.032*	
H16C	1.0347	0.1695	0.8575	0.032*	
C17	1.2390 (3)	0.3219 (2)	0.75437 (9)	0.0233 (4)	
H17A	1.1158	0.3097	0.7193	0.035*	
H17B	1.3224	0.2295	0.7665	0.035*	
H17C	1.3525	0.3914	0.7345	0.035*	
C18	0.6287 (3)	0.81640 (18)	0.90074 (8)	0.0141 (3)	
C19	0.8187 (3)	0.8637 (2)	0.95129 (9)	0.0188 (4)	
H19A	0.9064	0.7789	0.9727	0.028*	
H19B	0.7415	0.9159	0.9888	0.028*	
H19C	0.9291	0.9265	0.9245	0.028*	
C20	0.4410 (3)	0.72805 (19)	0.94254 (8)	0.0164 (3)	
H20A	0.3226	0.6979	0.9096	0.025*	
H20B	0.3625	0.7869	0.9773	0.025*	
H20C	0.5184	0.6431	0.9674	0.025*	
C21	0.4985 (3)	0.95322 (19)	0.86854 (9)	0.0197 (4)	
H21A	0.3753	0.9272	0.8361	0.029*	
H21B	0.6123	1.0145	0.8423	0.029*	
H21C	0.4250	1.0053	0.9067	0.029*	

### Atomic displacement parameters (Å^2^)

**Table:**

	*U*^11^	*U*^22^	*U*^33^	*U*^12^	*U*^13^	*U*^23^
S1	0.0224 (2)	0.0166 (2)	0.0123 (2)	−0.00013 (16)	−0.00189 (15)	0.00089 (15)
O1	0.0269 (7)	0.0187 (7)	0.0178 (6)	−0.0001 (5)	−0.0059 (5)	−0.0022 (5)
O2	0.0291 (7)	0.0214 (7)	0.0161 (6)	−0.0030 (5)	−0.0045 (5)	−0.0001 (5)
O3	0.0184 (6)	0.0173 (6)	0.0118 (5)	0.0002 (5)	0.0019 (4)	0.0032 (5)
N1	0.0214 (7)	0.0164 (8)	0.0147 (6)	0.0038 (6)	0.0009 (5)	0.0005 (6)
C1	0.0240 (8)	0.0151 (9)	0.0164 (8)	0.0056 (7)	−0.0031 (6)	−0.0053 (7)
C2	0.0233 (8)	0.0145 (9)	0.0149 (8)	0.0050 (7)	−0.0009 (6)	−0.0034 (6)
C3	0.0278 (9)	0.0192 (10)	0.0135 (8)	0.0066 (7)	−0.0025 (6)	−0.0030 (7)
C4	0.0301 (9)	0.0202 (10)	0.0131 (7)	0.0047 (7)	0.0015 (7)	0.0012 (7)
C5	0.0236 (9)	0.0190 (9)	0.0176 (8)	0.0021 (7)	0.0029 (7)	0.0012 (7)
C6	0.0217 (8)	0.0140 (9)	0.0134 (7)	0.0051 (7)	0.0010 (6)	−0.0015 (6)
C7	0.0193 (8)	0.0175 (9)	0.0143 (7)	0.0010 (7)	−0.0013 (6)	0.0004 (6)
C8	0.0156 (7)	0.0154 (9)	0.0135 (7)	−0.0001 (6)	−0.0003 (6)	−0.0001 (6)
C9	0.0154 (7)	0.0163 (9)	0.0143 (7)	0.0008 (6)	0.0003 (6)	−0.0035 (6)
C10	0.0129 (7)	0.0115 (8)	0.0153 (7)	−0.0007 (6)	−0.0012 (6)	−0.0017 (6)
C11	0.0123 (7)	0.0132 (8)	0.0122 (7)	−0.0018 (6)	−0.0013 (5)	0.0000 (6)
C12	0.0121 (7)	0.0125 (8)	0.0135 (7)	−0.0013 (6)	−0.0003 (5)	−0.0020 (6)
C13	0.0152 (7)	0.0126 (8)	0.0162 (8)	0.0014 (6)	−0.0009 (6)	0.0004 (6)
C14	0.0154 (7)	0.0125 (8)	0.0172 (8)	0.0013 (6)	−0.0008 (6)	−0.0016 (6)
C15	0.0145 (8)	0.0162 (9)	0.0242 (9)	0.0035 (7)	−0.0031 (6)	−0.0011 (7)
C16	0.0190 (8)	0.0137 (9)	0.0312 (9)	−0.0008 (7)	−0.0021 (7)	0.0000 (7)
C17	0.0285 (9)	0.0186 (10)	0.0224 (9)	0.0108 (8)	−0.0003 (7)	−0.0045 (7)
C18	0.0155 (7)	0.0120 (8)	0.0147 (7)	0.0009 (6)	0.0011 (6)	−0.0014 (6)
C19	0.0186 (8)	0.0194 (9)	0.0191 (8)	−0.0016 (7)	0.0014 (6)	−0.0061 (7)
C20	0.0141 (7)	0.0173 (9)	0.0180 (8)	−0.0001 (6)	0.0020 (6)	−0.0027 (7)
C21	0.0238 (9)	0.0144 (9)	0.0202 (8)	0.0068 (7)	0.0032 (7)	−0.0017 (7)

### Geometric parameters (Å, °)

**Table:**

S1—C6	1.7634 (16)	C12—C13	1.395 (2)
S1—C7	1.8239 (17)	C12—C18	1.542 (2)
O1—C1	1.321 (2)	C13—H13	0.9500
O1—H1O	0.850 (10)	C14—C17	1.538 (2)
O2—C1	1.230 (2)	C14—C16	1.538 (2)
O3—C11	1.3817 (18)	C14—C15	1.542 (2)
O3—H3O	0.8400	C15—H15A	0.9800
N1—C5	1.337 (2)	C15—H15B	0.9800
N1—C6	1.342 (2)	C15—H15C	0.9800
C1—C2	1.471 (3)	C16—H16A	0.9800
C2—C3	1.394 (2)	C16—H16B	0.9800
C2—C6	1.415 (2)	C16—H16C	0.9800
C3—C4	1.380 (3)	C17—H17A	0.9800
C3—H3	0.9500	C17—H17B	0.9800
C4—C5	1.385 (2)	C17—H17C	0.9800
C4—H4	0.9500	C18—C21	1.536 (2)
C5—H5	0.9500	C18—C19	1.540 (2)
C7—C8	1.505 (2)	C18—C20	1.539 (2)
C7—H7A	0.9900	C19—H19A	0.9800
C7—H7B	0.9900	C19—H19B	0.9800
C8—C9	1.386 (2)	C19—H19C	0.9800
C8—C13	1.387 (2)	C20—H20A	0.9800
C9—C10	1.393 (2)	C20—H20B	0.9800
C9—H9	0.9500	C20—H20C	0.9800
C10—C11	1.414 (2)	C21—H21A	0.9800
C10—C14	1.541 (2)	C21—H21B	0.9800
C11—C12	1.405 (2)	C21—H21C	0.9800
			
C6—S1—C7	100.24 (8)	C17—C14—C10	111.47 (13)
C1—O1—H1O	109.8 (18)	C16—C14—C10	110.37 (13)
C11—O3—H3O	126.6	C17—C14—C15	105.58 (13)
C5—N1—C6	118.43 (15)	C16—C14—C15	110.68 (14)
O2—C1—O1	122.91 (17)	C10—C14—C15	111.98 (14)
O2—C1—C2	122.32 (15)	C14—C15—H15A	109.5
O1—C1—C2	114.77 (15)	C14—C15—H15B	109.5
C3—C2—C6	117.92 (17)	H15A—C15—H15B	109.5
C3—C2—C1	120.55 (15)	C14—C15—H15C	109.5
C6—C2—C1	121.54 (15)	H15A—C15—H15C	109.5
C4—C3—C2	120.04 (16)	H15B—C15—H15C	109.5
C4—C3—H3	120.0	C14—C16—H16A	109.5
C2—C3—H3	120.0	C14—C16—H16B	109.5
C3—C4—C5	117.91 (16)	H16A—C16—H16B	109.5
C3—C4—H4	121.0	C14—C16—H16C	109.5
C5—C4—H4	121.0	H16A—C16—H16C	109.5
N1—C5—C4	123.84 (17)	H16B—C16—H16C	109.5
N1—C5—H5	118.1	C14—C17—H17A	109.5
C4—C5—H5	118.1	C14—C17—H17B	109.5
N1—C6—C2	121.87 (15)	H17A—C17—H17B	109.5
N1—C6—S1	115.97 (12)	C14—C17—H17C	109.5
C2—C6—S1	122.16 (14)	H17A—C17—H17C	109.5
C8—C7—S1	107.24 (11)	H17B—C17—H17C	109.5
C8—C7—H7A	110.3	C21—C18—C19	107.13 (14)
S1—C7—H7A	110.3	C21—C18—C20	106.49 (13)
C8—C7—H7B	110.3	C19—C18—C20	110.50 (13)
S1—C7—H7B	110.3	C21—C18—C12	111.61 (13)
H7A—C7—H7B	108.5	C19—C18—C12	109.74 (13)
C9—C8—C13	119.07 (14)	C20—C18—C12	111.26 (14)
C9—C8—C7	120.60 (15)	C18—C19—H19A	109.5
C13—C8—C7	120.31 (15)	C18—C19—H19B	109.5
C8—C9—C10	122.24 (15)	H19A—C19—H19B	109.5
C8—C9—H9	118.9	C18—C19—H19C	109.5
C10—C9—H9	118.9	H19A—C19—H19C	109.5
C9—C10—C11	116.84 (15)	H19B—C19—H19C	109.5
C9—C10—C14	120.15 (14)	C18—C20—H20A	109.5
C11—C10—C14	122.99 (14)	C18—C20—H20B	109.5
O3—C11—C12	116.25 (13)	H20A—C20—H20B	109.5
O3—C11—C10	121.08 (14)	C18—C20—H20C	109.5
C12—C11—C10	122.67 (14)	H20A—C20—H20C	109.5
C13—C12—C11	117.06 (14)	H20B—C20—H20C	109.5
C13—C12—C18	120.13 (14)	C18—C21—H21A	109.5
C11—C12—C18	122.80 (14)	C18—C21—H21B	109.5
C8—C13—C12	122.10 (15)	H21A—C21—H21B	109.5
C8—C13—H13	118.9	C18—C21—H21C	109.5
C12—C13—H13	118.9	H21A—C21—H21C	109.5
C17—C14—C16	106.52 (15)	H21B—C21—H21C	109.5
			
O2—C1—C2—C3	176.46 (16)	C9—C10—C11—O3	−179.75 (14)
O1—C1—C2—C3	−4.1 (2)	C14—C10—C11—O3	1.8 (2)
O2—C1—C2—C6	−3.6 (3)	C9—C10—C11—C12	0.3 (2)
O1—C1—C2—C6	175.85 (15)	C14—C10—C11—C12	−178.16 (15)
C6—C2—C3—C4	−0.1 (2)	O3—C11—C12—C13	−179.46 (13)
C1—C2—C3—C4	179.83 (16)	C10—C11—C12—C13	0.5 (2)
C2—C3—C4—C5	−0.1 (3)	O3—C11—C12—C18	1.9 (2)
C6—N1—C5—C4	−0.3 (3)	C10—C11—C12—C18	−178.11 (14)
C3—C4—C5—N1	0.3 (3)	C9—C8—C13—C12	0.6 (2)
C5—N1—C6—C2	0.0 (2)	C7—C8—C13—C12	179.43 (15)
C5—N1—C6—S1	−179.54 (12)	C11—C12—C13—C8	−1.0 (2)
C3—C2—C6—N1	0.2 (3)	C18—C12—C13—C8	177.73 (15)
C1—C2—C6—N1	−179.77 (15)	C9—C10—C14—C17	3.8 (2)
C3—C2—C6—S1	179.70 (12)	C11—C10—C14—C17	−177.85 (15)
C1—C2—C6—S1	−0.2 (2)	C9—C10—C14—C16	−114.38 (17)
C7—S1—C6—N1	−1.21 (15)	C11—C10—C14—C16	64.0 (2)
C7—S1—C6—C2	179.23 (14)	C9—C10—C14—C15	121.83 (16)
C6—S1—C7—C8	178.80 (11)	C11—C10—C14—C15	−59.8 (2)
S1—C7—C8—C9	85.94 (17)	C13—C12—C18—C21	2.6 (2)
S1—C7—C8—C13	−92.91 (16)	C11—C12—C18—C21	−178.80 (15)
C13—C8—C9—C10	0.3 (2)	C13—C12—C18—C19	−116.01 (16)
C7—C8—C9—C10	−178.57 (15)	C11—C12—C18—C19	62.6 (2)
C8—C9—C10—C11	−0.7 (2)	C13—C12—C18—C20	121.40 (16)
C8—C9—C10—C14	177.77 (15)	C11—C12—C18—C20	−60.00 (19)

### Hydrogen-bond geometry (Å, °)

**Table:**

*D*—H···*A*	*D*—H	H···*A*	*D*···*A*	*D*—H···*A*
O1—H1o···O2^i^	0.85 (1)	1.79 (1)	2.640 (2)	179 (3)

Symmetry codes: (i) −*x*, −*y*+1, −*z*+1.
